# An increase in reports of acute flaccid paralysis (AFP) in the United Kingdom, 1 January 2018–21 January 2019: early findings

**DOI:** 10.2807/1560-7917.ES.2019.24.6.1900093

**Published:** 2019-02-07

**Authors:** The United Kingdom Acute Flaccid Paralysis (AFP) Task Force

**Affiliations:** 1 Members of the UK AFP task force are listed at the end of the article.

**Keywords:** Acute flaccid paralysis, acute flaccid myelitis, enterovirus D68, United Kingdom, public health, EV-D68

## Abstract

During 2018, the United Kingdom experienced an increase in reports of cases of acute flaccid paralysis (AFP). As at 21 January 2019, 40 cases had been identified with a peak in October 2018. The increase was temporally associated with an upsurge in enterovirus (EV) D68 activity. Enterovirus was detected in 15 cases, mainly from respiratory tract samples; nine were typed as EV-D68. A national task force has been established and investigations are ongoing.

In October 2018, Public Health England (PHE) observed an increase in routine EV-D68 laboratory detections. PHE and other United Kingdom (UK) national public health agencies reminded clinicians of the potential respiratory and neurological associations of EV-D68 infection and the requirements for appropriate microbiological investigations including exclusion of poliomyelitis. In November 2018, PHE began to receive reports of acute flaccid paralysis (AFP). A national task force was established to investigate the apparent increase. Here, we describe the preliminary epidemiological, clinical and microbiological features of cases as at 21 January 2019.

## Acute flaccid paralysis investigation

### Case definition

A clinical case of AFP was defined as an individual of any age presenting with acute onset of flaccid paralysis affecting one or more limbs, not explained by a non-infectious cause with onset date since 1 January 2018. A probable case of acute flaccid myelitis (AFM) was defined as any person with symptoms of AFP and a cerebrospinal fluid (CSF) pleocytosis (white cell count (WCC) > 5 cells/mm^3^). A confirmed AFM case was defined as any person with symptoms of AFP and a spinal cord lesion largely restricted to grey matter on magnetic resonance imaging (MRI) scanning (Table 1), similar to the definition used by the United States (US) Centers for Disease Control and Prevention (CDC) [1].

**Table 1:** Case definition and ascertainment of acute flaccid paralysis cases, United Kingdom, 1 January 2018–21 January 2019

Case definition			Number of cases
Acute flaccid paralysis	Clinical	Any person presenting with symptoms of AFP not explained by a non-infectious cause	40
	Discarded	Not AFP OR AFP explained by a non-infectious cause	0
Poliomyelitis	Poliomyelitis-confirmed	AFP case in whom poliovirus was detected	0
	Pending	AFP case with inadequate specimens or samples not yet tested and/or 60 day follow-up not completed	2
	Poliomyelitis-discarded	AFP case where poliovirus infection was unlikely after expert review based on clinical, epidemiological and virological information	38
Acute flaccid myelitis	Probable	Any person with symptoms of acute flaccid limb paralysis AND CSF showing pleocytosis (WCC > 5 cells/mm^3^)	7
	Confirmed	Any person with symptoms of acute flaccid limb paralysis AND An MRI showing a spinal cord lesion largely restricted to grey matter spanning ≥ 1 spinal segments	9
	Pending	Any person with symptoms of acute flaccid limb paralysis AND An MRI unavailable or results need clarification	19
	Discarded	Any person with symptoms of acute flaccid limb paralysis AND MRI findings inconsistent with AFM	5
Non-polio enteroviral associated AFP	Confirmed	AFP case where non-polio enterovirus was detected in one or more sample (respiratory, stool, CSF)	15
	Negative	AFP case where non-polio enterovirus was not detected in appropriately timed samples (respiratory, stool, CSF taken within 14 days of illness onset)	8
	Pending	AFP case where specimens not taken or samples not yet tested	10
	Unexplained	AFP case where inadequate specimens available	7

AFM: Acute flaccid myelitis; AFP: acute flaccid paralysis; CSF: cerebral spinal fluid; MRI: magnetic resonance imaging; WCC: white cell count.

### Investigations in patients presenting with unexplained acute neurological symptoms and/or the presence of acute flaccid paralysis

Clinicians were instructed to perform specific investigations in adults and children presenting with unexplained acute neurological symptoms and/or the presence of AFP. For all AFP cases, rapidly notified to PHE, clinical and epidemiological information was collected, appropriate laboratory investigation (including exclusion of poliomyelitis) were advised with emphasis on respiratory and stool specimens being optimal samples for enterovirus detection. Local hospital laboratories were requested to send any EV-positive samples from AFP cases to the PHE Enteric Virus Unit (London, UK) for typing, including detection and confirmation of EV-D68 infection by EV-D68-specific reverse transcription (RT)-PCR and picornavirus VP1 sequencing. All cases of clinical or confirmed AFP/AFM also required two unadulterated stool samples, collected 24–48h apart, to be submitted to the PHE Polio Reference Service (London, UK) for exclusion of poliovirus infection by virus isolation.

The UK task force gathered detailed demographic and epidemiological information including recent travel history, polio vaccine history, clinical and radiographic information using a standardised questionnaire. The available clinical, epidemiological, laboratory and radiographic information of each case was reviewed by the investigators to determine case classification status, with particular focus on whether the case was poliomyelitis-confirmed, poliomyelitis-compatible or discarded (Table 1). In addition, each AFP case was reviewed to ascertain whether they were a probable, confirmed or discarded AFM case (Table 1) [1].

**Figure 1:**
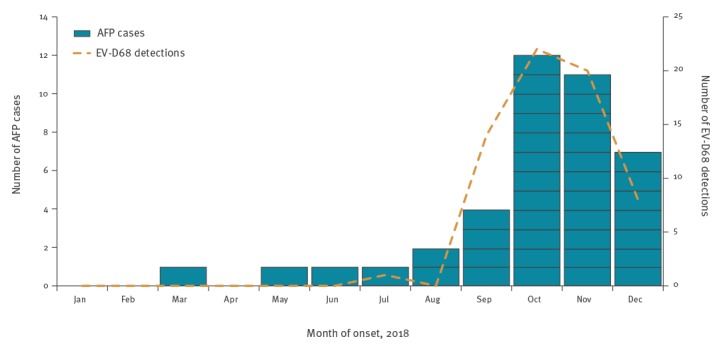
Number of acute flaccid paralysis cases (n = 40) and enterovirus D68 detections (n = 65) by month, United Kingdom, 1 January–31 December 2018

AFP: acute flaccid paralysis; EV-D68: enterovirus D68.

 As at 21 January 2019, 40 clinical AFP cases, scattered across the UK, had been notified to PHE, all with onset of symptoms occurring between 1 January 2018 and 31 December 2018 (Table 2). Six sporadic cases of AFP were reported January–August, followed by a rapid rise in cases during September with numbers peaking in October before then declining (Figure 1). The temporal pattern of AFP cases by week of onset coincided with the overall number of EV-D68 positive detections (from both respiratory and neurological cases) by week of sampling by the PHE reference laboratory (Figure 1).

**Table 2:** Characteristics of reported acute flaccid paralysis and acute flaccid myelitis cases, United Kingdom, 1 January–31 December 2018 (n = 40)

Case characteristics	AFP case (N= 40)	AFM case confirmed (n = 9)^a^	AFP case with EV-D68 detected (n = 9)
**Age group (years)**			
< 5	22	3	7
5–19	5	0	1
20–39	6	3	1
≥ 40	7	3	0
**Sex**			
Male	21	7	6
Female	19	2	3
**Recent travel history (< 1 month)**			
None	25	6	6
Europe	8	2	3
Outside Europe – low risk	1	1	0
Outside Europe – high risk^b^	1	0	0
Unknown	5	0	0
**Polio vaccine history**			
Fully vaccinated	36	8	9
Unknown	4	1	0
**NPEV laboratory result (upper respiratory tract specimen)**			
EV-D68	9	2	9
Rhinovirus	1	0	0
Coxsackie B1	1	1	0
EV-C104	1	0	0
EV untyped	3	0	0
EV/rhinovirus not detected	25	6	0
**Recent acute upper respiratory tract illness**			
Yes	22	4	6
No	18	5	3
**Limb weakness**			
Single	8	1	1
Two–three	11	2	1
All	20	6	7
**ICU admission**			
Yes	15	4	4
No	7	1	2
Unknown	18	4	3
**Assisted ventilation**			
Yes	12	3	4
No	11	0	2
Unknown	17	6	3

AFM: acute flaccid myelitis; AFP: acute flaccid paralysis; EV: enterovirus; ICU: intensive care unit; NPEV: non-polio enteroviruses.

^a^ Nine AFM cases of 40 AFP cases.

^b^ Countries where high risk of contracting polio infection.

Poliovirus was excluded in all but two AFP cases (pending the outcome of the ongoing investigation). Nine cases were confirmed AFM, seven were probable, five were discarded and as at 21 January 2019, 19 are pending further clinical and imaging details to classify status (Table 1). Non-polio enteroviruses were detected in 15 AFP cases to date; EV-D68 was detected in nine of those. A range of other picornaviruses were detected e.g. human rhinovirus, Coxsackie B1 and EV-C104, with enterovirus remaining untyped in two AFP cases; further characterisation is underway (Table 2).

EV-D68 RNA was detected in respiratory tract samples (nasopharyngeal aspirates, nose and throat swabs, sputum, bronchoalveolar lavage fluid and endotracheal aspirates) from the nine EV-D68-associated AFP cases; two of which also had EV-D68 RNA detected in faecal samples. Of 14 EV-positive cases with available dates, 12 had at least one sample taken within 2 weeks of illness onset, compared with 6 of 15 for EV-negative cases.

**Figure 2:**
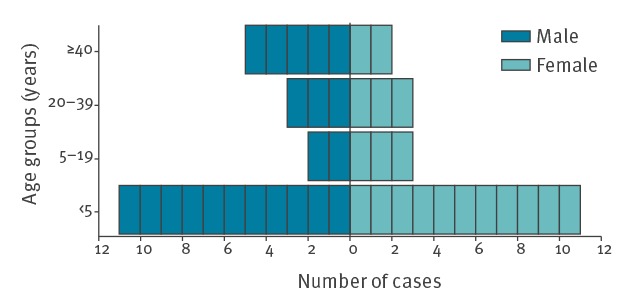
Number of acute flaccid paralysis cases by age and sex, United Kingdom, 1 January to 31 December 2018 (n = 40)

Approximately half of the AFP clinical cases (22/40) and the majority of the EV-D68 confirmed AFP cases (7/9) were aged 5 years or less (Figure 2 and Table 2). Although, the proportion of AFP cases were similar by sex, seven of nine confirmed AFM cases were male (Table 2). The majority (34/35) of AFP cases with information available had no relevant recent travel history, with only one case having recently returned from an area with a risk of contracting poliomyelitis.

Of 40 clinical AFP cases, 22 reported recent acute respiratory tract illness before onset of neurological symptoms. Half developed AFP in all four limbs, as did 7/9 confirmed EV-D68 cases. Of 22 AFP cases, 15 required admission to intensive care unit (ICU) and 12 needed assisted ventilation (Table 2). By 21 January 2019, eight AFP cases have been followed up 60 days after onset of symptoms, with three reported to still have a considerable neurological deficit.

## Discussion

The United Kingdom (UK) has observed an increase in reports of AFP cases, with many cases diagnosed as AFM. The US first reported unexpected clusters of AFM in 2014 [2]. Cases typically present clinically as poliomyelitis-like paralysis, affecting one or more limbs, with no apparent sensory loss and characteristic grey matter findings on MRI. The increase in AFM reports was temporally associated with increased circulation of EV-D68, a common EV infection that seems to circulate biennially in late summer and autumn, which was linked with severe acute respiratory and neurological illness in 2014 and 2016 in Europe, Asia and North America [3-6].

These cases in 2018 occurred primarily among pre-school age children, with approximately half of the cases reported with global limb paralysis and requiring respiratory support. Poliovirus infection has been excluded in all fully investigated cases as at 21 January 2019, with investigation pending for two cases. The pathogens most frequently isolated from samples were non-polio EVs, found in over a third of cases, with EV-D68 being the most frequent type. Active investigation identified many of the cases with CSF and MRI information available to be AFM, with classical MRI and/or CSF findings. The UK situation is very similar to that described recently in the US with almost identical findings [2,7,8], with an increase of cases with polio-like symptoms temporarily associated with circulation of EV-D68.

The current upsurge in reported cases is unexpected. Each year the UK reports only a small number (< five per year) of AFP cases to the World Health Organization (WHO) as part of national poliomyelitis surveillance; this is known to underestimate the true incidence of AFP. There are common alternative diagnoses for poliomyelitis, such as Guillain–Barré syndrome (GBS) and transverse myelitis (including AFM), but these are rarely investigated and reported for poliomyelitis surveillance. Between July 1991 and June 1994 active surveillance of AFP in children was conducted through the British Paediatric Surveillance Unit with 120 cases reported in total, of which 68 were GBS, four were confirmed as poliomyelitis [9], but only six had a diagnosis of transverse myelitis (data not shown). This suggests that the current upsurge in AFM cases is more than anticipated. Further work is required to understand the baseline epidemiology of AFP/M including interrogation of hospital records.

Our investigation has highlighted the clinical importance of reporting AFP and excluding poliomyelitis as an explanation. Although poliomyelitis has historically been responsible for most cases of infection-related flaccid paralysis, non-polio enteroviruses (NPEV) are well recognised to be associated with AFP/AFM [10-12]. We found that NPEV were detected in respiratory and/or faecal samples (rather than the CSF) for most cases who had adequate clinical material taken. The majority of NPEV were characterised as EV-D68 and positive samples were primarily from the respiratory tract. EV-D68 was not detected in any CSF sample, and only rarely reported globally [8]. Our findings emphasise the importance of correct and timely (within 2 weeks of onset) sample collection to ensure poliomyelitis can be excluded as a diagnosis, as well as to identify whether cases are co-infected with NPEV [13]. Such investigations are critical to better understand the aetiology of this condition.

There has been considerable debate whether EV-D68, which is recognised to be an almost ubiquitous infection in children, causes AFP/AFM [14]. Although the virus is rarely detected in the CSF, it is frequently found in adequate samples from the respiratory tract. We demonstrated a clear temporal association, with the rise in AFP/AFM cases and EV-D68 detections in the population (most of whom will have had respiratory presentations). Furthermore, EV-D68 was detected in the respiratory samples of almost a quarter of cases. Some researchers suggest recent genetic changes in the EV-D68 virus may have increased neurotropism and thus its ability to cause acute neurological disease in a very small proportion of those who have been infected, potentially in combination with host-genetic factors [15,16], although that hypothesis has recently been contested [17]. Work with mouse models has implicated EV-D68 in the aetiology of acute myelitis, but also suggested a potential protective role for EV-D68 immune sera [18]. Further study is required, however, to understand the potential role of EV-D68 in AFP/AFM and identify effective interventions to treat and ultimately prevent this condition.

In response to the apparent rise in AFP/AFM cases, the UK has created an AFP task force including clinical and laboratory working-groups. The aim of the group is to: strengthen AFP surveillance; ascertain whether there has been a true increase in incidence of AFP in the population; determine the aetiology of these cases, in particular the potential contribution of EV, especially EV-D68; systematically characterise the illness and long-term sequelae; and to increase awareness of optimal investigation and management of cases [19]. The group also aims to act as a focal point for national and international collaboration and to share widely the findings from this ongoing investigation as they emerge.

## The UK AFP task force

Members of the UK AFP task force: PHE: Mary Ramsay (chair), Jake Dunning, Sarah Foulkes, Jamie Lopez, Antoaneta Bukasa, Nalini Iyanger, Maria Zambon, Kevin Brown, Daniel Bradshaw, Shamez Ladhani, Vanessa MacGregor, Richard Pebody, Matthew Donati, Julia Stowe; Public Health Wales: Chris Williams; PHANI: Michael Devine ; Health Protection Scotland: Arlene Reynolds, Diogo Marques, Jim McMenamin; Edinburgh Specialist Virology Centre: Kate Templeton; Guys and St Thomas’ Trust: Ming Lim.
